# Lipoid Proteinosis presenting as beaded papules of the eyelid: report of three cases

**DOI:** 10.1186/s12886-021-01802-z

**Published:** 2021-01-13

**Authors:** Zhenyu Wei, Antoine Labbe, Qingfeng Liang

**Affiliations:** 1 Beijing Key Laboratory of Ophthalmology and Visual Sciences, Beijing Institute of Ophthalmology, Beijing Tongren Eye Center, Beijing Tongren Hospital, Capital Medical University, 100005 Beijing, China; 2 Quinze-Vingts National Ophthalmology Hospital, IHU FOReSIGHT, Paris, France; 3 Versailles Saint-Quentin-en-Yvelines University, Versailles, France; 4 Institut de la Vision, IHU FOReSIGHT, Sorbonne Université, INSERM, CNRS, 17 rue Moreau, F-75012 Paris, France

**Keywords:** Case report, Lipoid proteinosis, Eyelid disease, Extracellular matrix gene 1

## Abstract

**Background:**

Lipoid proteinosis (LP) is a rare multisystem inherited disease. We report here in three LP cases with beaded papules of the eyelid. Their clinical presentations, histological characteristics, and genetic findings are described and discussed.

**Case presentation:**

A 12-year-old boy reported to our hospital with a complaint of ocular irritation, redness, and tearing for two years. He had a history of hoarseness since childhood. His younger brother (5 years old) also complained of hoarseness. Another patient, a 26-year-old woman, described many beaded papules on the edge of her eyelids since age 11 years. She additionally reported hoarseness since 4 years of age. Careful slit-lamp examination in these cases revealed waxy beaded papules on the margins of both eyelids and mild conjunctival congestion. Physical examination showed irregular, rugged scars on their facial skin. Genetic analysis showed the mutation located in exon 6 of the ECM1 gene.

**Conclusions:**

Three LP cases first diagnosed by ophthalmologists are presented. The presence of eyelid papules should prompt the ophthalmologist to pay close attention to the patient’s voice. If there is a definite history of hoarseness, these patients should undergo gene sequence analysis. If necessary, otorhinolaryngology and dermatology consults may help confirm the diagnosis. Treatment is primarily symptomatic to improve patients’ quality of life.

## Background

 Lipoid proteinosis (LP), first described by Urbach and Wiethe in 1929, is an autosomal recessive genodermatosis caused by mutations in the extracellular matrix gene 1 (ECM1) on chromosome 1q21 [1]. LP varies in clinical manifestations and severity, usually presenting as hoarseness in early childhood, with subsequent mucocutaneous lesions [2]. Neurological complications have been also described [2]. Here, we report three cases of LP patients with beaded papules of the eyelid, along with oropharyngeal and skin manifestations.

## Case presentation

### Case 1-2

 A 12-year-old boy (case 1) was referred to our department with complaints of ocular irritation, redness, and tearing for 2 years. He had a history of hoarseness since childhood and complained of recurrent large ulcerations on his tongue, as well as fragile skin with bullous lesions that appeared after minimal trauma. His younger brother (5 years old, case 2) had no complaints other than hoarseness. They all denied a previous history of surgery or medication. Their parents and close relatives had no similar history.

**Fig. 1:**
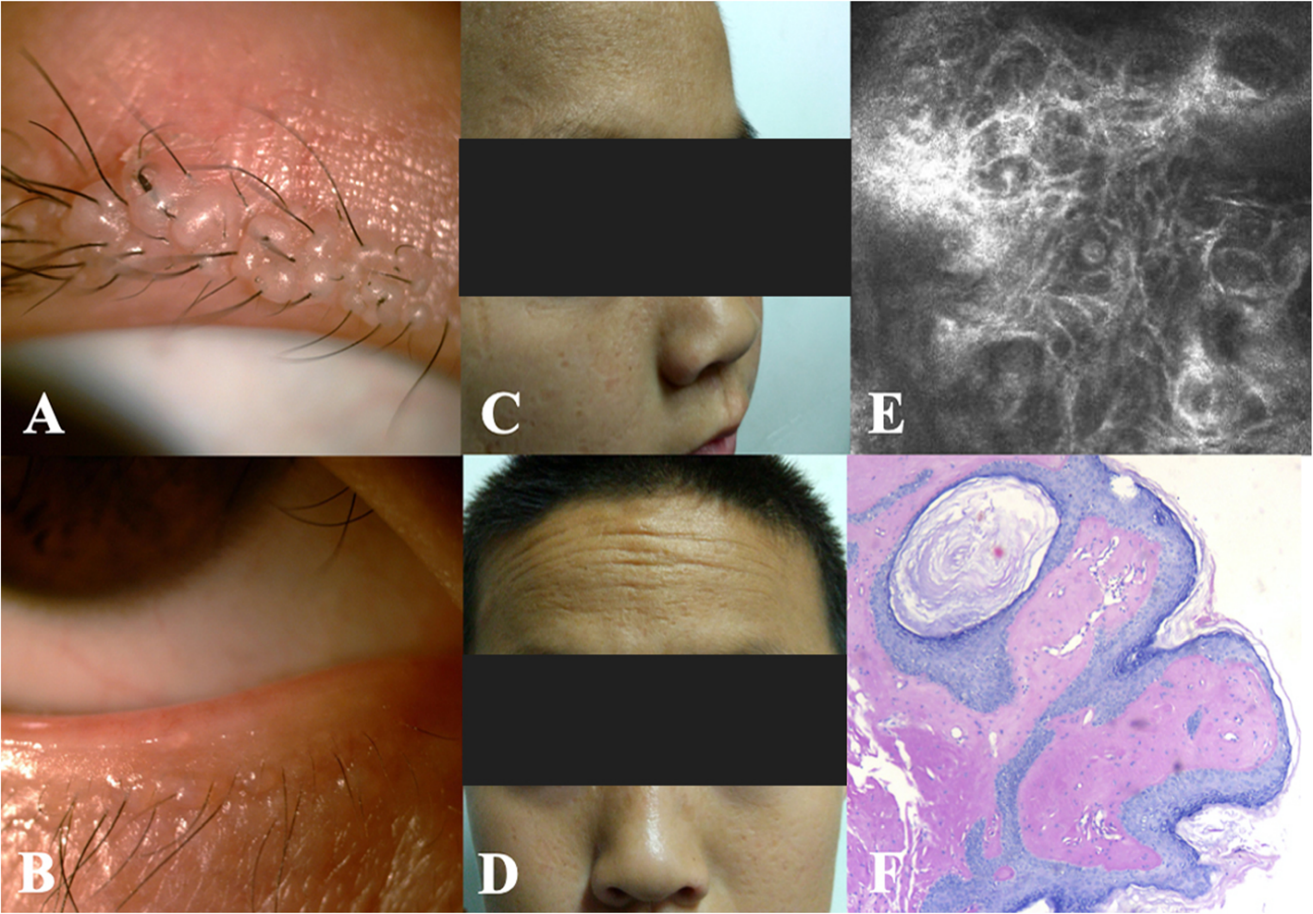
Clinical features and histological findings of lipoid proteinosis in cases 1–2. **a**, **b**, waxy beaded papules on the margins of both eyelids and conjunctival congestion. **c**, **d**, irregular, rugged facial scarring and yellowish plaques and fine lines on the forehead. **e**, highly reflective fiber-like substance intricately arranged under the epithelial cells on *in vivo* confocal microscopy (800×). **f**, homogeneous eosinophilic hyaluronic material infiltrating around sweat glands and capillaries, associated with epidermal thinning (hematoxylin and eosin staining, 400×)

Slit-lamp examination revealed waxy beaded papules on the margins of both eyelids and mild conjunctival congestion in both cases (Fig. 1a, b). Physical examination showed some irregular, rugged scars on the facial skin, yellowish plaques and fine lines on their foreheads (Fig. 1c, d). In vivo confocal microscopic (IVCM) analysis of the eyelids in case 1 showed highly reflective fibrous deposition under the epithelial cells (Fig. 1e).

A biopsy of an eyelid papule was performed in case 1. Periodic acid-Schiff staining showed infiltration of an eosinophilic hyaluronic substance into many areas of connective tissue. Similar deposits were also found around sweat glands and capillaries, associated with epithelial thinning (Fig. 1f). Direct laryngoscopy combined with histologic examination showed epithelial dysplasia of the laryngeal and epiglottal mucosa.

**Fig. 2:**
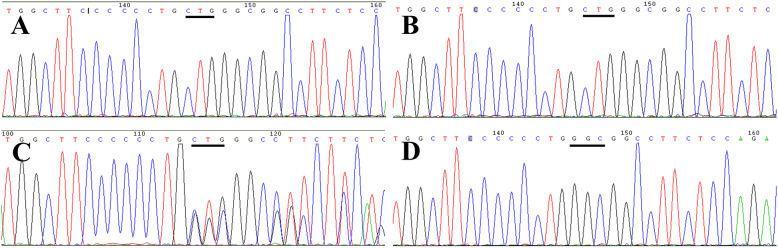
Pedigree (case 1 & 2) and sequencing results. **a**, **b**, homozygous CTG insert nucleotide 506 to 508 in exon 6 of the ECM1 gene in case 1 and case 2. **c**, same recessive heterozygous mutation in the parents. **d**, wild type nucleotides in this portion of the DNA sequence

Blood samples from the two affected cases and their parents were sent for gene sequence analysis. The two patients had the same homozygous CTG insert nucleotide 506 to 508 (c.506_508dupCTG) in exon 6 of the ECM1 gene (NM_004425.4) (Fig. 2a, b). Their parents had a similar heterozygous mutation at the same site, although they lacked the phenotype (Fig. 2c).

 The final diagnosis for the two boys was LP. The differential diagnosis included amyloidosis, hyalinosis, and erythropoietic protoporphyria. In response to the patient’s symptoms, lubricant eye drops were prescribed. We also informed the patients that, if necessary, the papular eyelid lesions could be surgically removed.

### Case 3

A 26-year-old woman who was bothered by the appearance of her eyelid margin with many beaded papules presented to our department. The hyperkeratotic papular lesions had appeared 15 years previously and worsened within the past 4 years. Her parents and close relatives had no similar history. The patient denied any history of prior surgery or medication.

**Fig. 3:**
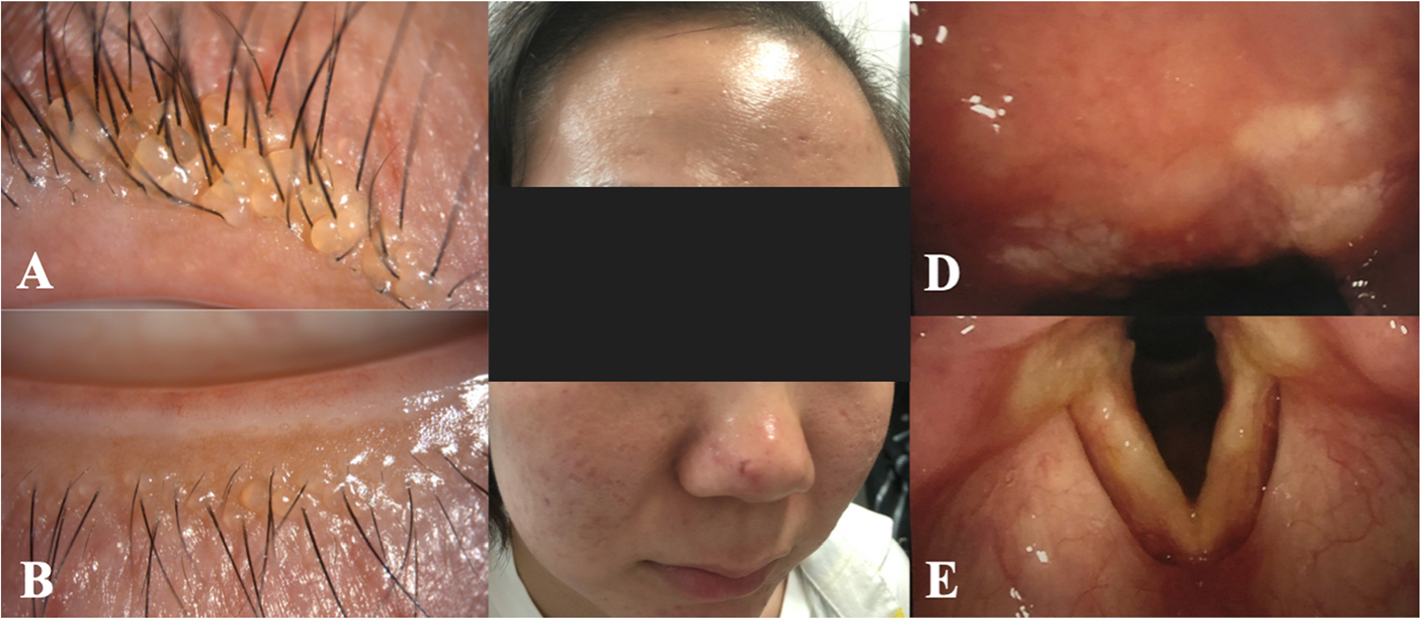
Clinical features of lipoid proteinosis in case 3. **a**, **b**, beaded papules on the eyelids in case 3. **c**, multiple rugged facial scars. **d**, **e**, pale yellow substance deposition in the oropharynx and bilateral vocal cords

The papular lesions of the upper and lower eyelids of both eyes were clustered closely together, forming beaded structures (Fig. 3a, b). In addition, she reported hoarseness since the age of 4 years. There was no history of similar symptoms among her family members. Cutaneous examination revealed waxy skin and erosive cutaneous lesions (Fig. 3c). Direct laryngoscopy showed deposition of a pale-yellow substance in the oropharynx and vocal cords, and movement of the vocal cords was restricted (Fig. 3d, e).

**Fig. 4:**
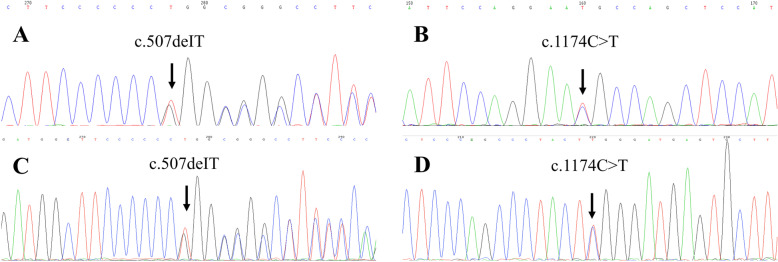
Pedigree (case 3 and her parents) and sequencing results**.** **a**, **b** patient’s genetic mutations: T base missing at nucleotide 507 and C base replacing T base at nucleotide 1174. **c**, the same genetic mutation was found in the patient’s mother. **d**, the same suspected pathogenic gene detected in her father

Genetic analysis showed a deleted T at nucleotide 507 (c.507deIT) in exon 6 of the ECM1 gene (NM_004425.4) (Fig. 4a). Analysis of the parents showed that this gene originated from her mother (Fig. 4c). Her father showed another suspected pathogenic gene in the same exon, with C replacing T at nucleotide 1174 (c.1174C > T) (Fig. 4b, d).

The woman’s final diagnosis was LP. The differential diagnosis excluded amyloidosis, hyalinosis, and erythropoietic protoporphyria. In order to smooth the patient’s ocular surface, we recommended lubricants. We advised her that if she wanted to improve her hoarseness, vocal cord surgery could be performed.

## Discussion and conclusions

Lipoid proteinosis is a phenotypically heterogeneous condition with variable clinical features due to infiltration of the skin and multiple organs by a hyaline-like material. Patients typically present in early childhood with vocal hoarseness and skin and mucous membrane changes characterized by yellowish waxy areas affecting the face. The presence of a row of beaded papules along the eyelid margin, known as moniliform blepharitis, is also thought to be pathognomonic. Infiltration of the upper airway can induce dysphagia and, in severe cases, respiratory obstruction. Epilepsy and neuropsychiatric abnormalities may also appear in children [3].

Clinical diagnosis is confirmed by histological findings on biopsy of cutaneous and mucous membrane lesions, showing disruption and/or duplication of the basement membrane along with deposition of hyaline material at the dermo-epidermal junction, papillary dermis and surrounding capillaries, and around adnexal epithelia. We have provided an IVCM image, which has rarely been used to detect LP. The similarities between the biopsy and IVCM image may suggest an alternative method of detecting eyelid disease. In addition, head CT and MRI examinations are used to detect intracranial calcifications in severe cases.

The molecular basis of LP has recently been elucidated, and it appears to result from mutations in the gene encoding extracellular matrix protein 1 (Ecm1, *ECM1*; OMIM 602,201). *ECM1* protein is expressed in the skin, mucosa, and several other tissues, including the placenta, heart, liver, small intestine, lungs, kidneys, and endothelial cells. *ECM1* can stimulate blood vessel endothelial cell proliferation and influence the differentiation of keratinocytes. *ECM1* promotes binding of collagen IV and laminin and acts as a biological glue by binding glycosaminoglycans to fibrillar protein growth factors [4]. Abnormal *ECM1* function results in reduced production of normal collagen, with abnormal interactions with perlecan, MMP-9, fibulin, and laminin [5]. *ECM1* protein may play a role in wound healing, scarring, and aging.

To date, at least 47 different mutations in the *ECM1* gene have been reported in more than 50 unrelated patients with LP [6]. Frameshift and nonsense mutations have been described throughout the gene, with exons 6 and 7 being the most common locations. Mutations in these locations appear to have genotype-phenotype relevance. Patients with exon 7 mutations display slightly milder clinical features, while mutations in exon 6 (as in our cases) result in a more severe phenotype [7].

LP lacks effective treatment. Fortunately, with its slow progression and stable symptoms in adulthood, LP does not generally affect lifespan. It has been reported that oral dimethyl sulfoxide [8], D-penicillamine [8], and ettretinate [9] can alleviate symptoms involving the larynx and facial skin, but the efficacy of these treatments remains controversial. For skin lesions, skin treatments, avoidance of potential skin trauma to minimize scarring, and scar removal may be performed. To treat vocal hoarseness, the pale-yellow deposits on the surface and edges of the vocal cords may be removed surgically [10]. Ophthalmologists can make an early diagnosis of LP upon observation of the typical eyelid manifestations. If the disorder does not affect eyelid movement, the beaded papules along the eyelids should not be removed, and observation is the better option.

To conclude, when examining patients with eyelid papules, any voice or skin changes should be carefully noted. With a definite history of hoarseness, these patients should be sent for gene sequence analysis. If necessary, otorhinolaryngology and dermatology consults may help confirm the diagnosis. For LP, treatment is primarily symptomatic to improve patients’ quality of life.

## Data Availability

Data sharing is not applicable to this article as no datasets were generated or analyzed during the current study.

## Abbreviations

LP: Lipoid proteinosis
ECM1: Extracellular matrix gene 1
IVCM: In vivo confocal microscopy
